# A Successful Coronary Artery Bypass Operation with Intermittent Factor VIII Administration in a Hemophilia A Patient Who Was Admitted Due to Acute Myocardial Infarction: A Rare and Difficult Case

**DOI:** 10.4274/tjh.galenos.2018.2018.0271

**Published:** 2019-05-03

**Authors:** Ulaş Serkan Topaloğlu, Rıfat Özmen, Recep Civan Yüksel, Murat Çetin, Gülşah Akyol

**Affiliations:** 1 Kayseri City Training and Research Hospital, Clinic of Internal Medicine, Kayseri, Turkey; 2 Kayseri City Training and Research Hospital, Clinic of Cardiovascular Surgery, Kayseri, Turkey; 3 Erciyes University Faculty and Medicine, Department of Internal Medicine, Intensive Care Unit, Kayseri, Turkey; 4 Erciyes University Faculty and Medicine, Department of Cardiology, Kayseri, Turkey; 5 Kayseri City Training and Research Hospital, Clinic of Hematology, Kayseri, Turkey

**Keywords:** Hemophilia A, Coronary artery bypass surgery, Intermittent factor VIII administration

## To the Editor,

There is not a large study in the literature other than a few case reports and reviews about the procedure of coronary artery bypass grafting surgery planned for patients with hemophilia. An internationally accepted definitive algorithm that recommends an approach to these patients was not included in the guidelines.

**Figure 1:**
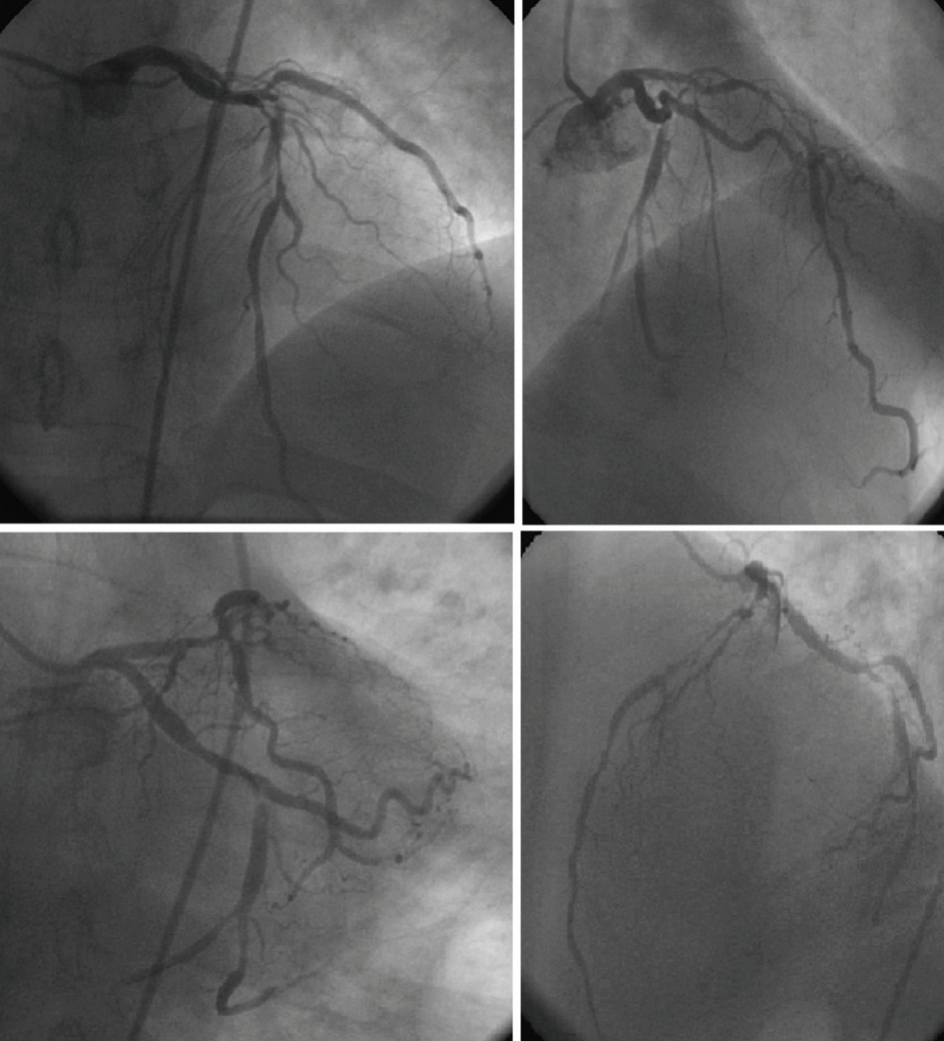
Stenosis: right coronary artery, 50%; left anterior descending artery, 80%; and circumflex coronary artery, 90%.

A 51-year-old male patient admitted in emergency service due to sudden and severe chest pain. He had no other medical history except hemophilia A. His electrocardiographic findings showed ST elevation in derivations II, III, and aVF. Troponin T at 0.19 ng/mL (normal: 0-0.1) was accepted as positive. The patient was admitted to the coronary intensive care unit with an initial diagnosis of acute inferior myocardial infarct and coronary angiography was urgently performed. Angiography revealed a moderate left ventricular ejection fraction (49%) with three occluded coronary arteries. The left anterior descending artery was critically stenotic up to 80%. The right coronary artery was stenotic up to 50%. The circumflex coronary artery was also stenotic up to 90% (Figure 1). The patient received 50 U/kg (4000 U) factor VIII (FVIII) after angiography, which he was not able to receive before angiography due to the urgency of the case. Thereafter, he received 25 U/kg (2000) FVIII twice a day for 3 days, and then 20 U/kg (1600 U) FVIII was given for the following 7 days at intervals of 12 h. No intervention was performed during angiography because of multi-vessel disease and bypass operation was decided. Among blood parameters tested during admission of the patient, the activated partial thromboplastin time (aPTT) was 51.4 s. The FVIII inhibitor test was negative. His childhood FVIII level was 11.3; thus, he was evaluated as having a mild case of hemophilia A.

Prior to the bypass operation, the patient received 50 U/kg (4000 U) FVIII replacement and was taken to the operation with an aPTT value of 45.6 s. The bypass operation was carried out with the same procedures as for non-hemophiliac patients including standard heparinization. In order to prevent disseminated intravascular coagulation during factor replacements of the patient, heparin was not used except for a pump procedure. After the patient was weaned from the cardiopulmonary pump, 50 U/kg (4000 U) bolus FVIII was administered. For the following 3 days, 25 U/kg (2000 U) FVIII was administered at intervals of 12 h. Thereafter, 20 U/kg (1600 U) FVIII was administered for 7 days at intervals of 12 h (Table 1). The patient has been followed for 3 years with routine controls. Within this period, he has had no serious medical problems except nosebleeds.

When the literature was analyzed, it was identified that continued infusion of FVIII was rarely administered in pre- and intraoperative periods [1,2]. Similar to the literature, we did not administer continued infusion of FVIII because we believed that thrombosis risk was more of an issue compared to the bleeding.

The World Hemophilia Federation recommends FVIII levels between 80% and 100% before and after major operations [3], but considering the urgency and thrombosis risk in our case we brought a different approach, addressing all disciplines responsible for the case and arriving at a consensus. We present our method in Table 1 as a recommendation. In this method, we administered 2x50 U/kg on the day of the operation (1 day), 2x25 U/kg for the following 3 days, and 2x20 U/kg for the following 7 days and we named it the “1-3-7 protocol”. Our protocol needs to be tested with further studies.

## Figures and Tables

**Table 1:**
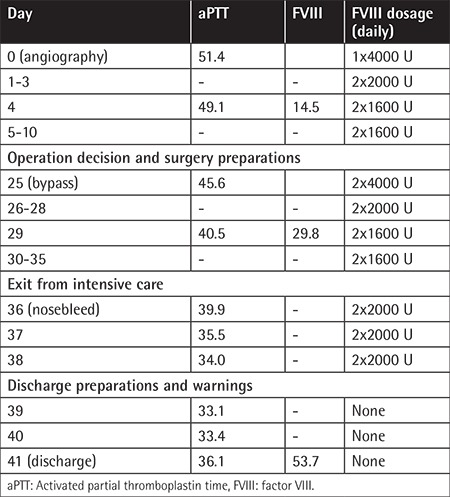
Daily total dosage of factor VIII and evaluation of activated partial thromboplastin time and factor VIII during the perioperative period.
